# Further Studies into Crack Growth in Additively Manufactured Materials

**DOI:** 10.3390/ma13102223

**Published:** 2020-05-12

**Authors:** Athanasios P. Iliopoulos, Rhys Jones, John G. Michopoulos, Nam Phan, Calvin Rans

**Affiliations:** 1 Computational Multiphysics Systems Laboratory, Center for Materials Physics and Technology, US Naval Research Laboratory, Washington, DC 20375, USA; athanasios.iliopoulos@nrl.navy.mil (A.P.I.); john.michopoulos@nrl.navy.mil (J.G.M.); 2 Centre of Expertise for Structural Mechanics, Department of Mechanical and Aerospace Engineering, Monash University, Clayton, Victoria 3800, Australia; 3 Structures Division, Naval Air Systems Command, Patuxent River, MD 20670, USA; nam.phan@navy.mil; 4 Faculty of Aerospace Engineering, Delft University of Technology, Kluyverweg 1, 2629 HS Delft, The Netherlands; C.D.Rans@tudelft.nl

**Keywords:** additive manufacture, aircraft sustainment, fatigue, Structures Bulletin EZ-SB-19-01, MIL-STD-1530D

## Abstract

Understanding and characterizing crack growth is central to meeting the damage tolerance and durability requirements delineated in USAF Structures Bulletin EZ-SB-19-01 for the utilization of additive manufacturing (AM) in the sustainment of aging aircraft. In this context, the present paper discusses the effect of different AM processes, different build directions, and the variability in the crack growth rates related to AM Ti-6Al-4V, AM Inconel 625, and AM 17-4 PH stainless steel. This study reveals that crack growth in these three AM materials can be captured using the Hartman–Schijve crack growth equation and that the variability in the various *da/dN* versus Δ*K* curves can be modeled by allowing the terms Δ*K_thr_* and *A* to vary. It is also shown that for the AM Ti-6AL-4V processes considered, the variability in the cyclic fracture toughness appears to be greatest for specimens manufactured using selective layer melting (SLM).

## 1. Introduction

The recent memo by the Under Secretary, Acquisition and Sustainment [1] enunciated that as of March 21, 2019, additive manufacturing (AM) is used to “enable the transformation of maintenance operations and supply chains, increase logistics resiliency, and improve self-sustainment and readiness”. This memo further stated that: “AM parts or AM repair processes can be used in both critical and non-critical applications. For all applications, the appropriate level of qualification, certification, and risk/safety evaluation must be completed by the appropriate engineering support activity”. 

This statement represents a significant development since, until recently, the focus has been on “low hanging fruit,” i.e., non-critical parts not affecting the safety of flight. However, as suggested in [2] and is reflected in [1,3] AM offers the potential for the “on-demand” manufacturing of structural parts, albeit with a life that may be less than the original design life but sufficient to ensure continued operational capability until a conventionally manufactured replacement part can be obtained. Structures Bulletin EZ-SB-19-01 [4] subsequently noted that to achieve this goal requires the development of analytical methods that are capable of assessing the damage tolerance of AM parts. This requirement is also echoed in [2,3,5,6,7,8,9,10]. To this end, [3] outlined an approach for assessing the potential for an AM replacement part to meet a limited life/durability requirement. Here, it should be noted that JSSG2006 [11] defines the term durability as the ability of the airframe to resist cracking (including stress corrosion and hydrogen-induced cracking), corrosion, thermal degradation, delamination, wear, and the effects of foreign object damage for a specified period of time. As such, the certification requirements associated with AM replacement parts are linked to the durability requirements outlined in [4,11,12,13,14].

This approach involved:

(i) Adopting the initial flaw assumptions, termed Equivalent Initial Damage Sizes (EIDS) in both Mil-STD-1530D [12] and the Joint Services Structural Guidelines [12], that as per [5] when performing a durability analysis for a life limited part an EIDS of at least 0.02 inch (0.508 mm). Here, it should be noted that EZ-SB-19-01 [4] and MIL-STD-1530D [12] adopt the definition of EIDS given in [13], viz: as an analytical characterization of the initial quality of the aircraft structure at the time of manufacture, modification, or repair. As such, the EIDS is not necessarily the physical size of the associated material discontinuity; see [11,13,14,15] for more details.
(ii) Using the *da/dN* versus Δ*K* curve for small cracks, as suggested by Lincoln and Melliere [14] when performing a durability analysis, to assess the “economic service life” of military aircraft. (The economic service life is defined in MIL-STD-130D [12], where the economic service life is linked to “service life limits less than the design requirement”. As such, the economic service life analysis methodology is relevant to AM replacement parts.)
(iii) Setting the “limited life” of the replacement part to be one-third of the computed life.

**Figure A1:**
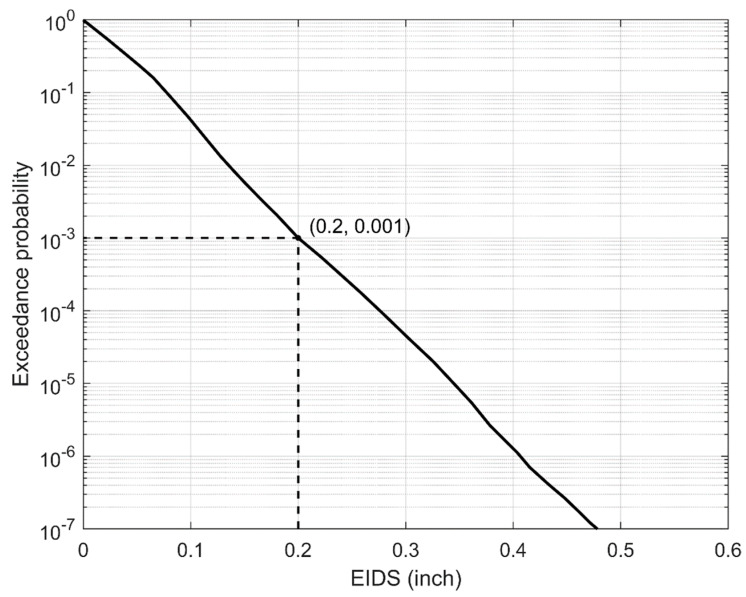
The exceedance probability curve for conventionally manufactured wrought aluminum from [5].

However, a more detailed examination of the data presented in [5,16,17], and to be consistent with MIL-STD-1530D [12], led to [3] suggesting that Step (iii) be replaced by setting the ‘limited life” of the replacement part to be at least half of the computed life. Here, as per [5], it was also noted that if the scatter in the materials data was too high, then the factor of 2 could be increased. As a result, this (modified) approach becomes similar to that subsequently proposed by Babish [5]. The specific differences between [3] and [5] are that [3]:

(a) Suggested EIDS of 0.02 inch (0.508 mm) for limited life parts; see Figure A1 in Appendix A of the present study. This size was based on the probability of exceeding the EIDS of 0.001.
(b) Babish [5] did not specify what crack growth curve should be used.

The subsequent USAF Structures Bulletin EZ-SB-19-01 [4], which established the requirements for the Durability and Damage Tolerance (DADT) certification of aircraft structural metallic parts fabricated from an additive manufacturing (AM) process, stated that the EIDS for durability crack growth analysis of durability critical and fatigue critical parts shall be based on a probability of exceeding the EIDS of 0.001, but not less than 0.01 inches (0.254 mm). The data given by Tiffany in [5] that were used to reveal that this requirement could lead to an EIDS of 0.02 inch (0.508 mm) are given in Appendix A. These data are not contained in EZ-SB-19-01 [4].

At this point, it should be noted that the choice of an EIDS of 0.02 inch (0.508 mm) is consistent with the EDIS values determined by an Airbus [7] of 0.251 mm for heat-treated parts manufactured using direct metal laser sintering (DMLS) and 0.448 mm for heat-treated parts manufactured using electron beam melting (EBM). It is also consistent with the statement by Airbus [8] that for “as-built” surfaces, the EIDS rarely exceeds 0.5 mm. Indeed, [9,10] both list a number of material discontinuity sizes that equate to approximately 0.5 mm. It should also be stressed that as explained in [4,11,12,13], the use of an EIDS plays a central role in establishing the durability/economic life of a part. Indeed, as shown in [3], moderately small changes in the EIDS value can result in significant changes in the computed life of an AM replacement part. It should also be stressed that as explained in [14,15], the value of the EIDS is dependent on the da/dN versus ΔK curve used. Using the da/dN versus ΔK curve as determined via tests on long cracks results in values that depend on the flight load spectrum [14,15]. This is why [14,15] stress the need to use the small crack da/dN versus ΔK curve when assessing the durability/economic life of a part.

**Figure 1:**
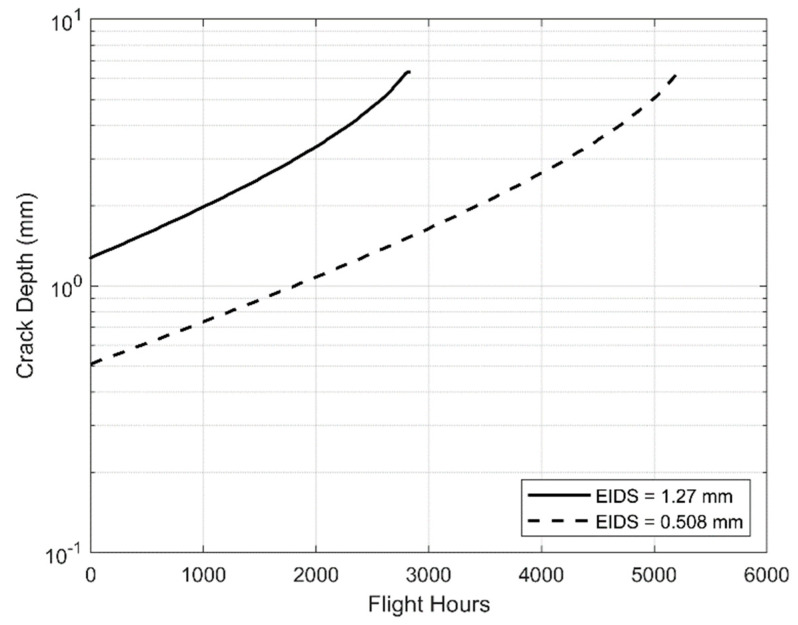
Influence of Equivalent Initial Damage Sizes (EIDS) on the computed fatigue life.

To illustrate the effect of using an EIDS of 0.508 mm, rather than the value of 1.27 mm used in [3], to determine the limited life of an AM part, consider the problem analyzed in [3], viz: crack growth in an additively manufactured Ti-6Al-4V specimen with a width of 80 mm and a thickness of 6.35 mm exposed to an industry-standard Combat Aircraft flight load spectrum (FALSTAFF) with a maximum stress in the spectrum of 396.5 MPa. This problem was selected because it approximates the crack propagation response at critical locations of the F/A-18 bulkhead [3]. Figure 1 presents the crack growth history computed in [3] using the da/dN versus ΔK curve associated with a small crack in AM Ti-6Al-4V and an EIDS of 1.27 mm. Figure 1 also presents the crack growth history computed using an EIDS of 0.508 mm. (It is interesting to note that Figure 1 also illustrates how, as noted above and in [15,18,19,20], when the small crack da/dN versus ΔK curve is used, the resultant crack growth histories are often approximately exponential.) Here, we see that using an EIDS of 0.508 mm increases the computed number of flight hours (Flt Hrs) to failure from approximately 2838 to 5199. 

Consequently, using an EIDS of 1.27 mm is very much more conservative than assuming an EIDS of 0.508 mm. Adopting the approach outlined in [4] would lead to a “durability” life, i.e., limited life of 2599 (=5199/2) Flt Hrs, or a life of 1419 (=2838/2) Flt Hrs if a fatigue critical analysis had been performed. If, as in [3], to account for the possibility of an increased scatter, a factor of 3 had been used, then these lives would reduce to 946 and 1733 Flt Hrs, respectively. Nevertheless, regardless of the size of the EIDS, or the safety factor used, the life of the part the life is still a significant proportion of the original design life of the airframe, viz: 6000 Flt Hrs. As such, it would be attractive for use as a replacement part.

It follows from MIL-STD-1530D [12] and EZ-SB-19-01 [4] that quantifying crack growth, understanding the variability in crack growth, including the effect of different build directions, the interaction between the surface roughness and manufacturing defects on or in proximity to the surface, and the ability to determine an upper bound on the crack growth curve are essential steps in the certification of additively manufactured replacement parts. Indeed, EZ-SB-19-01 [4] states that the accurate prediction of structural performance is the most difficult challenge facing AM materials. The MIL-STD-1530D [12] and EZ-SB-19-01 [4] requirement to perform a damage tolerance and durability analysis (DADT) requires a knowledge of the *da/dN* versus Δ*K* curves associated with AM materials. However, it is now known [3,16,17] that whereas for conventionally manufactured materials, the variability in the cyclic fracture toughness term *A* can be small, for AM materials, its variability can be quite large. Furthermore, the influence of this variability on the total life can be significant [3].

Similarly, it is also known that the interaction between surface roughness and manufacturing defects can also have a significant effect on fatigue life. (A summary of the importance of surface and subsurface defects in AM materials and their interaction with surface roughness is presented in [3].) In this context, it should be noted that although cracks generally initiate from surface defects [3], the stress concentration effect due to near-surface porosity/lack of fusion can combine with the stress concentration associated with a nearby rough surface and thereby increase both the likelihood of crack initiation and the subsequent crack growth rate. On the other hand it is also shown [3] that it can cause a surface-generated crack to change its path. This raises the problem of how to account for this interaction. This consideration was a factor in the recommendation given in [3] to (initially) adopt an EIDS of 1.27 mm. The specific words used in [3] were “it is suggested that an EIFS of 1.27 mm is sufficiently large in comparison to the length scales associated with both the surface roughness and the associated material discontinuities that their effect on the fatigue life of an AM part that is computed using this EIDS is likely to be minimal”. However, the introduction of EZ-SB-19-01 [4], and the data presented in [5]—that revealed for a durability analysis, an EIDS with a minimum size of 0.02 inch (0.508 mm) may be sufficient to ensure a probability of exceeding the EIDS of 0.001 (see Appendix A)—suggests that the value of 1.27 mm can be reduced. 

Returning to the critical question of the variability in the crack growth curves associated with AM materials, this paper builds on previous studies [3,16,17,21] and illustrates the degree of variability in the *da/dN* versus Δ*K* curves associated with AM Ti-6Al-4V materials that have been either heat-treated or HIPed (hot isostatic pressed). We also illustrate that provided there are sufficient test data, as is the case for crack growth in AM Ti-6Al-4V, a reasonably accurate representation of the associated upper bound curve and the small crack curve can be calculated using the Hartman–Schijve crack growth equation [3,15,16,17,18,19,20,21]. The present study also analyses crack growth in AM Inconel 625 and 17-4 PH stainless steel. It is shown that the crack growth in these particular additively manufactured materials can also be modeled using the Hartman–Schijve crack growth equation and that the variability of the relevant curves can be captured by allowing the terms Δ*K_thr_* and *A* to vary.

## 2. Materials and Methods

The studies analyzed in this paper are from publicly available peer-reviewed journals, reports, and other manuscripts. The bibliography consulted by the authors is based on peer-reviewed journal articles and conference proceeding papers. Most references in the present work are listed in WOS and SCOPUS and, others are available from the US Department of Defense DTIC website. There are also references from the US Federal Aviation Authority (FAA) and the U.S Navy’s data repositories. Keywords used in these searches were Additive Manufacturing, AM, durability, damage tolerance, Hartman–Schijve, small cracks, crack growth in operational aircraft, full-scale fatigue tests, and aircraft certification. The exceptions to this are the memo from the Under Secretary, Acquisition and Sustainment [1], and the USAF Structures Bulletin EZ-SB-19-01 [4], both of which are unclassified and have no release restriction. NAVAIR provided both of these documents. Keywords used in these searches were additive manufacturing, durability, damage tolerance, Hartman–Schijve, small cracks, crack growth in operational aircraft, full-scale fatigue tests, aircraft certification, etc. 

The main analyses methods used in the present paper are described in detail [17], and also in the subsequent sections.

## 3. Crack Growth in AM Ti-6Al-4V

The March 2012 edition of ASTM F2792-12a [22] defines additive manufacturing as “a process of joining materials to make objects from 3D model data, usually layer upon layer, as opposed to subtractive manufacturing methodologies”. This often involves using powders or materials in the form of a wire. The review paper [23] noted that while additive manufacturing offers the potential to economically fabricate customized parts with complex geometries, the mechanical behavior of these materials must be better understood before AM can be utilized for critical applications. This need is also described in MIL-STD 1530D and EZ-SB-19-01. The design, certification, and approval phases necessitate tools that enable the modeling of crack growth. The role of physical testing is to validate or calibrate the damage tolerance analysis. In this context, Cao, Zhang, Ryde, and Lados [24] presented an early review of cracking in AM Ti-6Al-4V, where fatigue thresholds associated with the different AM processes were carefully assessed. References [3,16,17,21] subsequently revealed that crack growth in a range of AM materials could be represented by the Hartman–Schijve (HS) variant of the NASGRO crack growth equation, viz:

*da*/*dN* = *D*(Δκ)*^p^* (1)

where *D* and *p* are constants, and the crack driving force is defined as per Schwalbe [25], viz:

Δ*κ* = (Δ*K* − Δ*K_thr_*)/(1 − (*K*_max_/*A*))^1/2^ (2)

where *A* is the cyclic fracture toughness and Δ*K_thr_* is the fatigue threshold. For Ti-6Al-4V, *D* = 2.79 × 10^−10^ and *p* = 2.12.

In the case of AM Ti-6Al-4V, Equation (1) was found [16,17] to hold irrespective of whether the AM process:

(a) was direct metal laser sintering (DMLS);
(b) was selective laser melt (SLM);
(c) was electron beam melting (EBM);
(d) was laser engineered net shaping (LENS), regardless of the process power level;
(e) invovled horizontal or vertical specimens,
(f) was followed by HiPing.

EZ-SB-19-01 [4] requires that the DADT data needed to certify AM applications should account for differences in variability or scatter compared to parts manufactured from wrought materials. In this context, it has been shown [16,17,21] that the variability in the *da/dN* versus Δ*K* curves associated with various AM processes can be captured, as per the variability in the growth of both long and small cracks in conventionally manufactured metals [15,18,19,20], by allowing for changes in the terms ∆*K_thr_* and *A*. Furthermore, as shown in [17], the small crack *da/dN* versus Δ*K* curve for LENS Ti-6Al-4V could be obtained from the long crack curves as per [15,18,19,20] by setting the fatigue threshold term Δ*K_thr_* to a small value: in this case, Δ*K_thr_* = 0.1 MPa √m.

**Figure 2:**
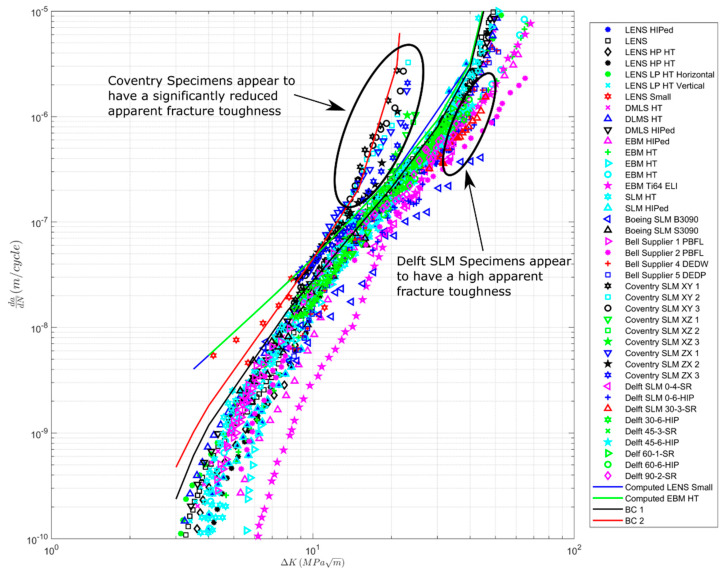
Variability in crack growth of AM Ti-6Al-4V specimens that have been Hipped or Heat-Treated (HT), data from [3,7,16,17,26,27,28,29,30,31].

To demonstrate the variability in the crack growth curves of AM materials, Figure 2 presents the *R* = 0.1 *da/dN* versus Δ*K* curves of a range of AM Ti-6Al-4V specimens that have been either HIPed or heat-treated (HT). The intention of this figure is not to present the data for each case but rather to illustrate the variation of the data due to each process used to manufacture the specimens distinct in terms of the overall relationship between the crack growth data of AM specimens. Several of the datasets shown in Figure 2 are also shown in [16,17], albeit not on the same plot. Figure 2 also contains plots of the Bell Helicopter study [26], which evaluated the effects of heat treatments on microstructure and properties of Ti-6Al-4V alloy fabricated by electron beam melting (EBM), Powder Bed Fusion Laser (PBFL), Directed Energy Deposition of Wire (by Electron Beam) (DEDW), and Directed Energy Deposition of Powder (by Laser) (DEDP). These curves are labeled Bell Supplier 1 PBFL, Supplier 2 PBFL, Supplier 4 DEDW, and Supplier 5 DEDP. Plots of the curves presented in [27] are labelled Boeing SLM S3090 and Boeing SLMS C3090. 

Figure 2 also presents a *da/dN* versus Δ*K* curves for:

(i) EBM Ti-6Al-4V ELI [28], and in Figure 2, the curve is labeled EBM Ti-64V ELI.
(ii) SLM manufactured Ti-6Al-4V [29], where the *R* = 0.1 crack growth curves were determined in the XY, XZ, and ZX directions, where X was the build direction. In this paper, these tests are labeled Coventry XY, Coventry XZ, and Coventry ZX.
(iii) SLM Ti-6Al-4V [30], where the effect of stress relief (SR) and stress relief plus HIPing (HIP) was evaluated on crack growth at angles of 0, 30, 45, 60, and 90 degrees to the build direction. The tests that were performed at R = 0.1 are labeled Delft-p-q, where p takes the values 0, 30, 45, 60, or 90 depending on the orientation of the specimen to the build direction, and q is the specimen number. Here, it should be noted that [30] had an error in the formulae used to determine the stress intensity factor K. Therefore, the *da/dN* versus Δ*K* curves given in [30] differed markedly from other studies. The mistake is corrected in the present paper. Details can be found in Appendix B.
(iv) The *R* = 0.1 SLM Ti-6Al-4V curves presented in [31], which are labeled SLM HT (heat-treated) and SLM HIP.

Figure 2 also contains:

(a) The curve “BC1”, which was computed using Equation (1) with *A* = 58 MPa √m and Δ*K_thr_* = 2.1 MPa √m.
(b) A curve “BC2”, which was computed using Equation (1) with *A* = 24.5 MPa √m and Δ*K_thr_* = 1.8 MPa √m.
(c) Small crack LENS *da/dN* - Δ*K* curves presented in [25], and the small crack growth curve computed in [17] with *A* = 58 MPa MPa √m and Δ*K_thr_* = 0.1 MPa √m.

Examining Figure 2, we see that SLM specimens have the most significant difference in the cyclic fracture toughness, with those specimens reported in [29] having the smallest value and those specimens reported in [30] having significantly larger toughness’s. Indeed, the cyclic fracture toughness associated with the tests reported in [30] were similar to those seen by Bell Supplier 2, who used Powder Bed Fusion Laser (PBFL). 

We also see that the curve BC2 appears to represent an upper bound on the tests reported in [29], and hence on all of the various AM Ti-6Al-4V *da/dN* versus Δ*K* curves. On the other hand, the curve BC1 appears to represent an approximate upper bound on all the tests other than the SLM tests reported in [29]. We also see that the scatter in the various curves is greater at high values of *da/dN* than for values near the fatigue threshold. This finding is consistent with that discussed in [16].

**Figure 3:**
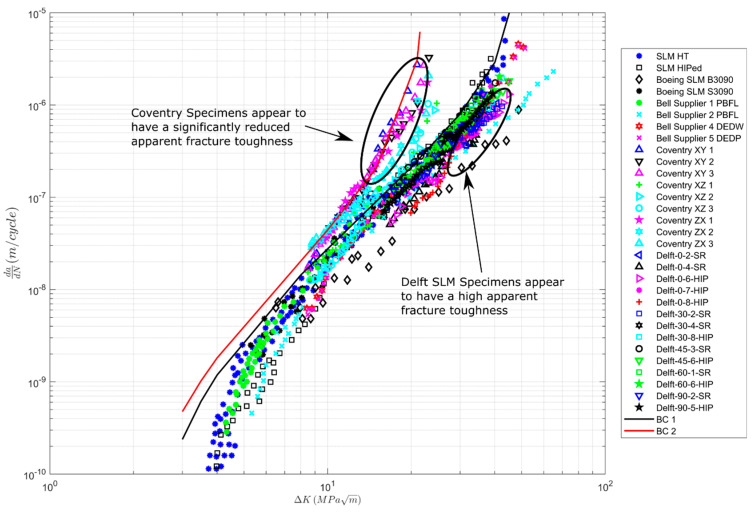
Variability in crack growth in selective layer melting (SLM) specimens that have been either Hipped or Heat-Treated (HT); the data are from [3,16,17,29,30,31].

**Figure 4:**
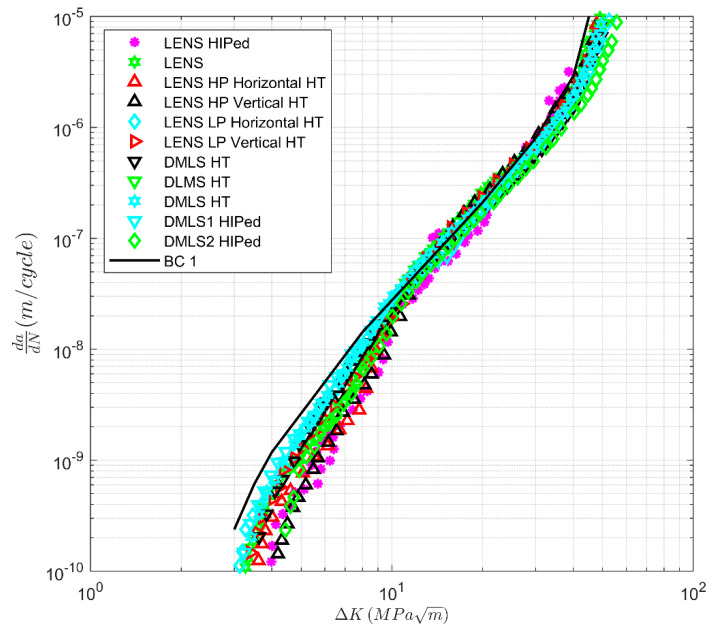
Variability in crack growth in laser engineered net shaping (LENS) and direct metal laser sintering (DMLS) specimens that have been either Hipped or Heat-Treated (HT); the data are from [3,16,17,28,33,34,35].

**Figure 5:**
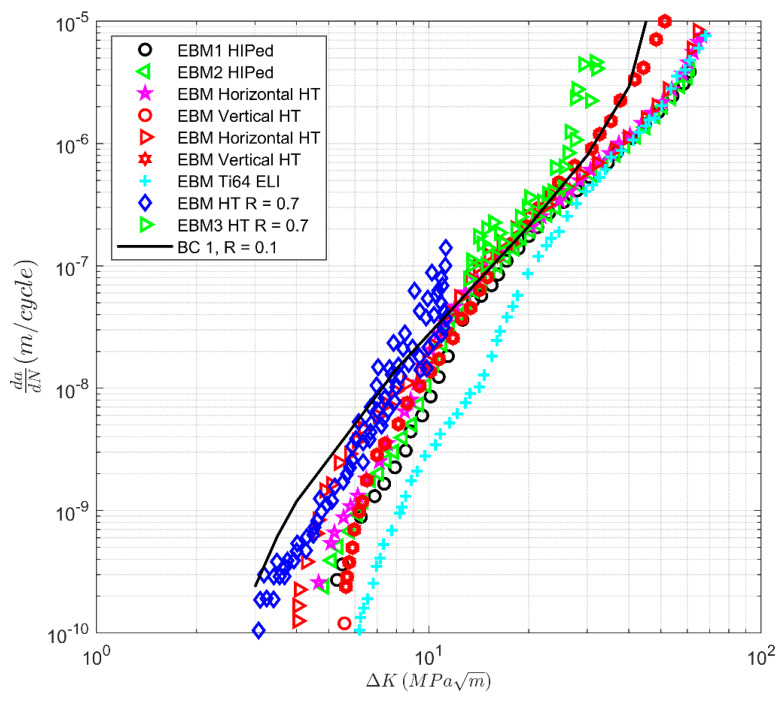
Variability in crack growth in electron beam melting (EBM) specimens that have been either Hipped or Heat-Treated (HT); the data are from [3,16,17,28,33,34,35].

To some extent, Figure 2 is a little misleading in that it compares a range of different AM processes. To clarify matters, Figure 3, Figure 4 and Figure 5 present the variability in the individual processes. Figure 4 and Figure 5 contain the curve BC1, and Figure 3 contains the curves BC1 and BC2. Here, we see that:

(1) The variability in the *R* = 0.1 *da/dN* versus Δ*K* curves at the near-threshold region associated with SLM specimens is comparable with that observed in conventional materials [16,17,32]. However, in Region III, the variability is higher than that seen in conventionally manufactured materials. This is due to instances when heat treatment, stress relief, and HIPing led to improvements in the fracture toughness; see Figure 3. The effect of the build direction on the *da/dN* versus Δ*K* curves appears to be small.
(2) The variability in the *da/dN* versus Δ*K* curves for *R* = 0.1 associated with LENS and DLMS specimens is similar with that observed in conventionally manufactured materials. Unlike the SLM specimens, there appears to be little variability in the cyclic fracture toughness; see Figure 4. For the LENS data, the effect of the build direction on the *da/dN* versus Δ*K* curves is not significant. No data on this effect for DLMS specimens were found.
(3) The variability in the *R* = 0.1 *da/dN* versus Δ*K* curves associated with EBM specimens is again similar to that of conventionally manufactured materials, and there appears to be little variability in the cyclic fracture toughness; see Figure 5. The effect of different build directions on the *da/dN* versus Δ*K* curves also appears to be small.
(4) We also see that the cyclic fracture toughness associated with LENS, DMLS, and EBM processes are higher than the values associated with the SLM specimen tests discussed in [29]. However, the cyclic fracture toughness’s are slightly lower than the values associated with the SLM specimen tests discussed in [30].

The fact that value of *A* = 58 MPa √m used to determine the curve BC1 in Figure 2, Figure 3, Figure 4 and Figure 5 is close to the value of *A* = 62 MPa √m used in [17] to study the potential for the use of AM Ti-6Al-4V as replacement parts suggests that LENS, EBM, and DMLS parts, when experiencing representative flight load spectra, would present a fatigue life that is sufficiently long for use as aircraft replacement parts. The potential for SLM specimens to have low cyclic fracture toughness also supports the proposal outlined in [17] to use fracture toughness measurements to evaluate the potential of the AM process to produce an acceptable replacement part.

To complete this study, Table 1 presents the values of the Δ*K*_thr_ and *A* for datasets that had values of *da/dN* that were close to the 10^−10^ m/cycle. Analysis of the table yields a mean Δ*K_thr_* of 3.46 (MPa √m), a standard deviation of approximately 0.88 (MPa √m), a mean *A* of 75.5 (MPa √m), and a standard deviation of approximately 18.0 (MPa √m). The values have been calculated in accordance with the approach described in [17].

### Small Cracks in AM Ti-6Al-4V 

For problems related to aircraft sustainment and to evaluate the life of parts where cracks arise and subsequently grow from material discontinuities, it is often necessary to use the small crack *da/dN* versus Δ*K* curve [13,14,15,19,20]. Indeed, the paper by Zhai et al. [35] suggested that the use of typical (long) crack growth *da/dN* versus Δ*K* curves to calculate the fatigue life of AM components may be non-conservative. As a result, [17] revealed that the small crack *da/dN* versus Δ*K* curves presented in [17] for AM Ti-6Al-4V could be estimated from the corresponding long crack curve by setting the threshold term Δ*K_thr_* to a small value. This finding is an extension of that reported in [15,18,19,20,36] for small crack assumptions in conventionally manufactured materials. 

**Figure 6:**
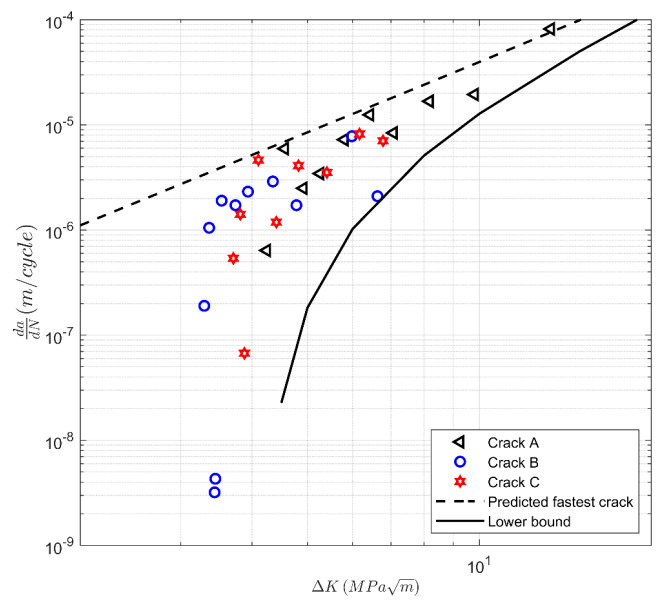
Variability in the crack growth curves observed in Hipped Ti-6Al-4V from [37].

Next, we investigate the validity of the hypothesis that by setting ΔK_thr_ to a small value, we can obtain a reasonable upper bound of the growth rate of small cracks in AM Ti-6Al-4V specimens. The variability can be estimated by allowing for variations in the term ΔK_thr_. To this end, Figure 6 shows the short crack *da/dN* versus Δ*K* curves presented in [37] together with the corresponding curves calculated using Equation (1) with D = 2.79 10^−10^ and p = 2.12. Reference [17] suggested that for the AM Ti-6Al-4V specimens studied in [35], a value of *A* = 128 MPa √m may be used. As such, the small crack equation for AM Ti-6Al-4V became

*da/dN* = 2.79 10^−10^ [(Δ*K* − Δ*K*_thr_)/(1 − *K*_max_/*A*)^1/2^]^2.12^ (3)

and, as per [15,18,19,20,36], to obtain an estimate of the crack growth rate related to the fastest-growing crack [17] set Δ*K*_thr_ = 0.1 MPa √m. This curve is also presented in Figure 6. In addition, Figure 6 contains the curve obtained using Equation (3) together with Δ*K*_thr_ = 4.2 MPa √m. Thus, it would appear that the results presented in Figure 6 support the hypothesis presented in [17] that Equation (3), with the appropriate fracture toughness term (*A*), yields reasonable first approximations for the *da/dN* versus Δ*K* curve and the variability in the crack growth curve related to the fastest-growing crack.

## 4. Crack Growth in Inconel 625

**Figure 7:**
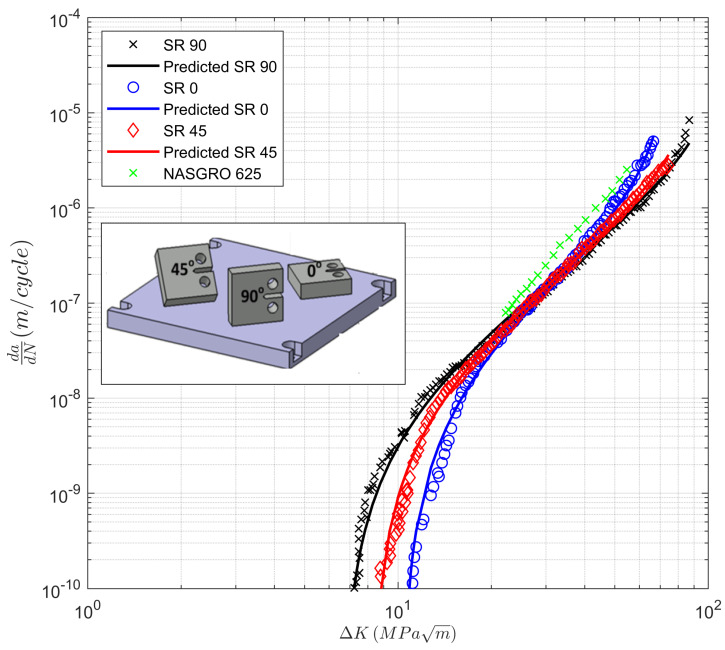
Crack growth curves for stress relieved (SR) laser powder bed fusion (LPBF) specimens cut from the 0, 45, and 90 directions, from [38].

It should be stressed that the findings discussed in Section 3 are specific to AM Ti-6Al-4V, and in particular, to the various AM processes analyzed. For example, unlike the data analyzed above, the *R* = 0.1 *da/dN* versus Δ*K* curves related to crack growth in stress relieved (SR) AM Inconel 625 [38] show a dependency on the build direction; see Figure 7. (Figure 7 also shows the *da/dN* versus Δ*K* curve, which is a conventionally manufactured (wrought) Inconel 625 taken from the NASGRO database).

The *da/dN* versus Δ*K* curves were also calculated using the Hartman–Schijve equation. The particular form of the equation was:

*da/dN* = 2.79 10^−10^ [(Δ*K* − Δ*K*_thr_)/(1 − *K*_max_/*A*)^1/2^]^1.99^ (4)

with *D* = 2.79 10^−10^, *p* = 1.92. The associated *da/dN* versus Δ*K* curves are also presented in Figure 7, where we observe an excellent agreement with the data. The associated values of *A* and Δ*K_thr_* are presented in Table 2. It should be noted that the value of *p* was also an output of the global optimization step described in [17].

**Figure 8:**
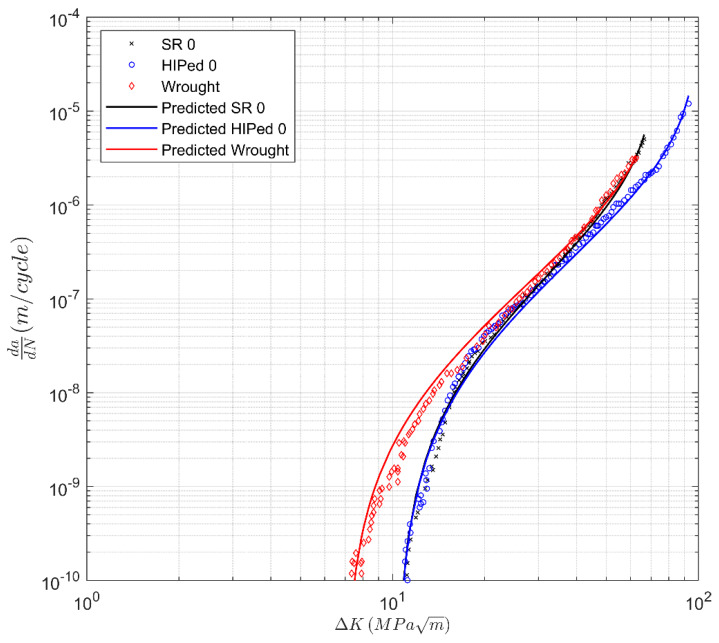
Measured and computed curves for stress relieved (SR) laser powder bed fusion. (LPBF), Hipped, and wrought Inconel 625 from [38].

Poulin et al. [38] also studied Inconel 625 specimens that were Hipped. The associated measured and computed *da/dN* versus Δ*K* curves obtained using Equation (4) are presented in Figure 8, where excellent agreement is observed. The values of *A* and Δ*K*_thr_ used in Figure 8 are given in Table 2.

## 5. Crack Growth in Additively Manufactured 17-4 PH Stainless Steel

**Figure 9:**
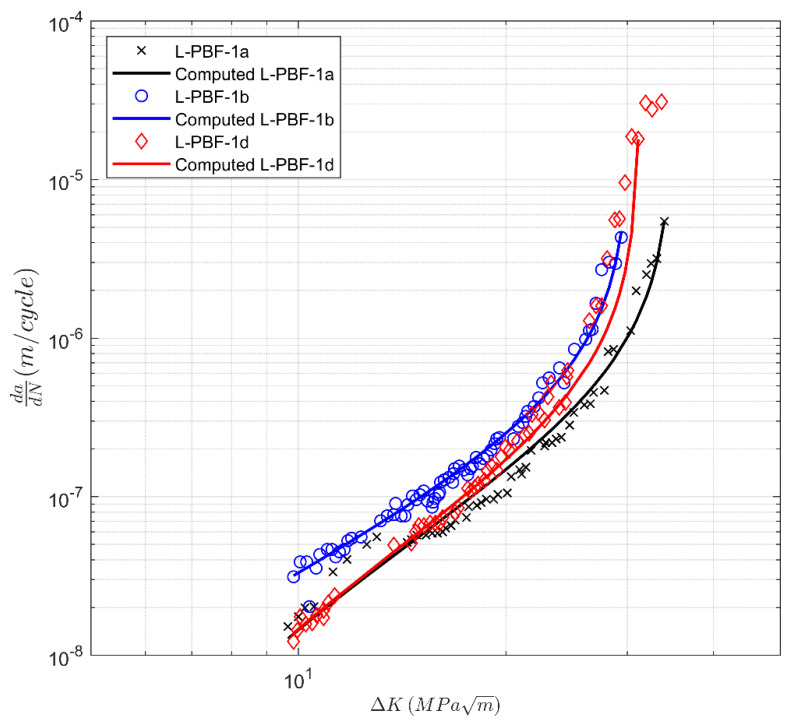
Crack growth curves for heat-treated laser powder bed fusion 17-4 PH stainless steel specimens tested in the 0° direction (Set 1) from [39]. The three last points of L-PVF-1d were not considered in the curve fitting.

**Figure 10:**
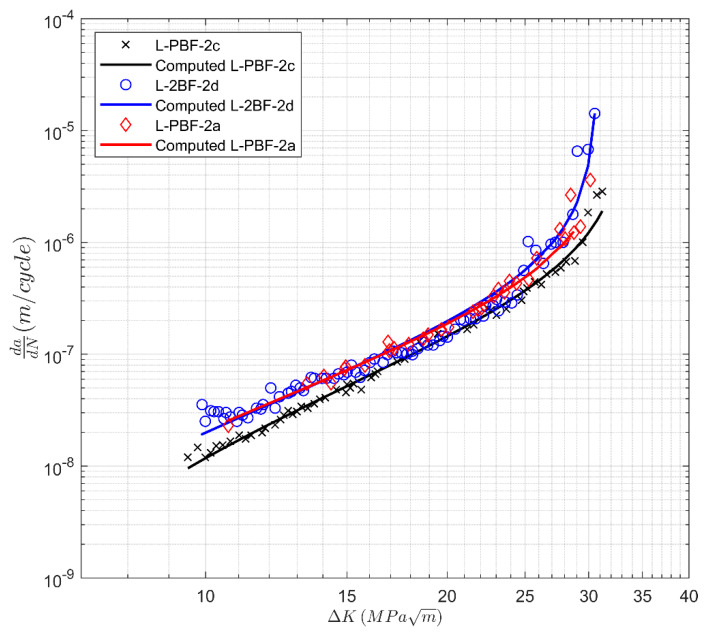
Crack growth curves for heat-treated laser powder bed fusion 17-4 PH stainless steel specimens tested in the 90° (Set 2) direction from [39].

Next, we consider the crack growth data presented in [39] for a heat-treated additively manufactured 17-4 precipitation hardening (PH) stainless steel (SS). These specimens were manufactured using a laser powder bed fusion (LPBF) system. This steel was labeled 17-4 PH Stainless Steel in our analysis. The CA-H900 heat-treatment process was used to increase the strength of the steel [39]. The CA-H900 heat-treatment process involved two steps. Specimens were heat-treated in solution at 1050 °C for 0.5 h and subsequently air-cooled to room temperature (i.e., Condition A (CA)). Then, the specimens were heated and kept at 482 °C for 1 hr and were air-cooled to room temperature (H900). Two sets of specimens were tested. Set 1 had the notch parallel to the build direction, and Set 2 had the notch perpendicular to the build direction. The specimens in these two sets were labeled L-PBF-1 and L-PBF-2, respectively. The resultant *R* = 0.1 *da/dN* versus Δ*K* curves are reproduced in Figure 9 and Figure 10. These figures reveal that in this instance, there is variability in both the fatigue threshold and in the cyclic fracture toughness. 

**Figure 11:**
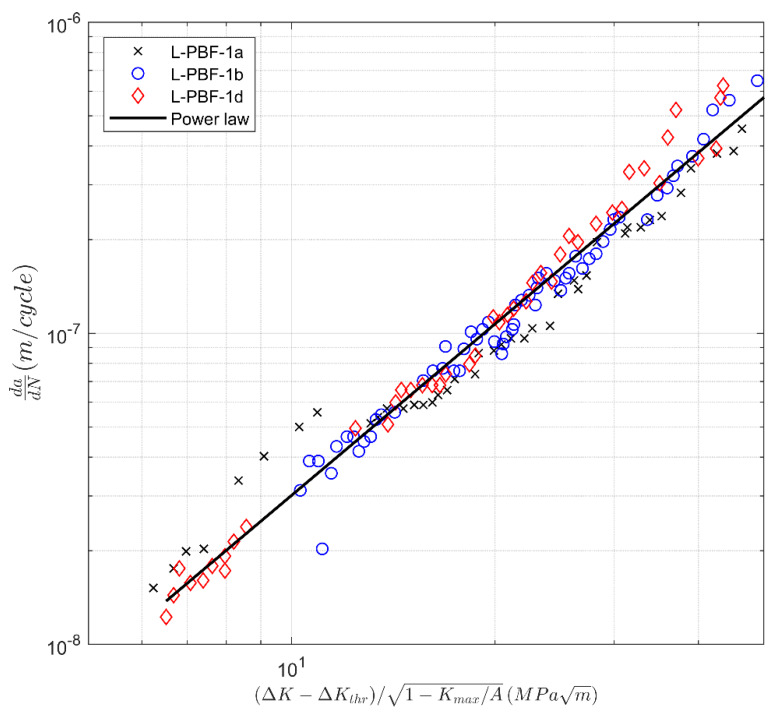
The *da/dN* versus Δ*K* curves for heat-treated laser powder bed fusion 17-4 PH stainless steel specimens tested in the 0° (Set 1) direction from [39]. This particular way of plotting collapses the computed curves into a single power law curve.

**Figure 12:**
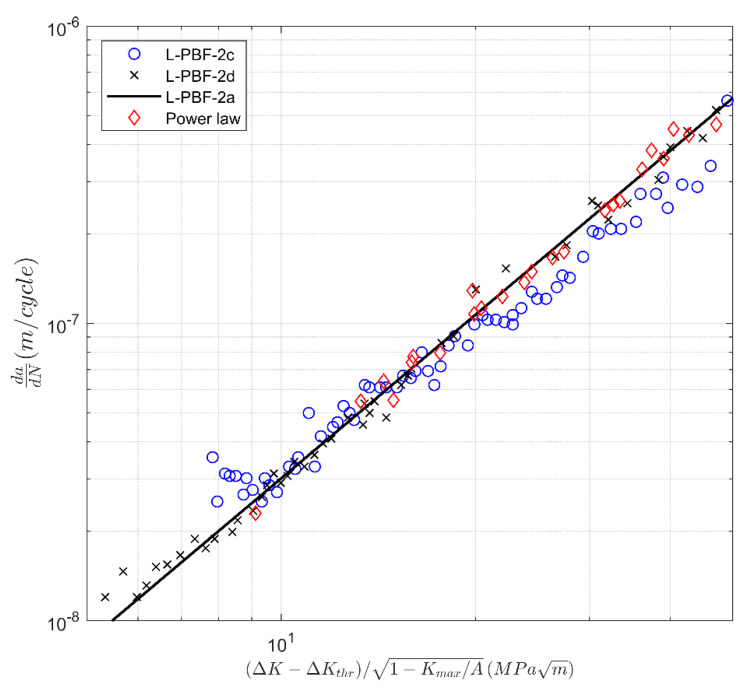
The *da/dN* versus Δ*K* curves for heat-treated laser powder bed fusion 17-4 PH stainless steel specimens tested in the 90° (Set 2) direction from [39]. This particular way of plotting collapses the computed curves into a single power law curve.

A plot of the associated *da/dN* versus Δκ curves are shown in Figure 11 and Figure 12. It should not come as a surprise that when plotted in this form and when allowing for the variability in the fatigue threshold Δ*K*_thr_ and *A*, the various crack growth curves essentially collapse onto the master curve:

*da/dN* = 4.46 10^−10^ [(Δ*K* − Δ*K*_thr_)/(1 − *K*_max_/*A*)^1/2^]^1.83^ (5)

This is an additional way of viewing the data, which may be beneficial when grouping together tests that have similar characteristics. The values of *A* and Δ*K*_thr_ used in Figure 9, Figure 10, Figure 11 and Figure 12 are given in Table 3.

## 6. Conclusions

As delineated in EZ-SB-19-01 [4], the ability to calculate crack growth and to account for the variability of the different AM processes is central to the certification of AM replacement parts. Consequently, the present paper addresses the variability resulting from various AM processes that have produced Ti-6Al-4V specimens. A comparison of the *da/dN* versus Δ*K* curves associated with SLM, LENS, DMLS, and EBM manufacturing processes, and the effect of HIPing and stress relief, as well as the impact of the build direction on the *da/dN* versus Δ*K* curves, has been presented. This study revealed the large variability seen in the cyclic fracture toughness associated with specimens fabricated using SLM. This variability was less pronounced in specimens fabricated using LENS, DMLS, and EBM processes. We also saw that specimens subjected to either HIPing and stress relief or just stress relief had similar *da/dN* versus Δ*K* curves and that the effect of different build directions on crack growth appeared to be relatively small. However, it should be stressed that these findings are specific to AM-produced Ti-6Al-4V specimens produced and post-processed by various AM processes.

It is further shown that the crack growth of both AM 17-4 PH stainless steel and AM Inconel 625 can be modeled using the Hartman–Schijve crack growth equation. In both instances, it is shown that the variability in the *da/dN* versus Δ*K* curves can be modeled by allowing for variability in the terms Δ*K_thr_* and *A*.

## Figures and Tables

**Table 1:** The values of the fatigue threshold (∆*K*_thr_) and the cyclic toughness (*A*) as determined from Figure 2, Figure 3, Figure 4 and Figure 5. Additional values can be found in [17].

Label	∆*K*_thr_ (MPa √m)	*A* (MPa √m)
EBM Ti64 ELI	5.65	101.7
EBM Vertical HT 1 (circle)	4.51	65.4
EBM Vertical HT 2 (star)	4.47	65.4
EBM1 HIPed	4.40	110.1
EBM2 HIPed	4.05	108.9
EBM Horizontal HT	3.75	98.9
LENS HP Vertical HT	3.60	62.2
DMLS2 HIPed	3.53	73.1
LENS HIPed	3.48	55.5
EBM Horizontal HT	3.19	92.3
LENS HP Horizontal HT	3.01	62.2
DMLS HT 1	2.94	68.3
LENS LP Vertical HT	2.77	63.1
LENS HT	2.70	60.8
LENS LP Horizontal HT	2.63	68.8
DMLS HT 3	2.60	63.3
DLMS HT 2	2.52	68.8
DMLS1 HIPed	2.52	69.3

**Table 2:** The values of the fatigue threshold (∆*K_thr_*) and the cyclic toughness (*A*) as determined from Figure 7 and Figure 8.

Label	∆*K_thr_* (MPa √m)	*A* (MPa √m)
Wrought	6.95	84.5
SR, 0 direction	10.3	82.4
SR, 45 direction	8.25	107.2
SR, 90 direction	6.65	128.4
Hipped	10.32	112.5

**Table 3:** The values of ∆*K*_thr_ and *A* as determined from Figure 11 and Figure 12.

Label	∆*K*_thr_ (MPa √m)	*A* (MPa √m)
L-PBF-1a	4.34	38.9
L-PBF-1b	1.37	33.7
L-PBF-1d	4.44	34.8
L-PBF-2a	4.98	37.4
L-PBF-2c	3.45	34.2

## Appendix A. The Exceedance Probability Curve

The exceedance probability curve given by Babish [5] is shown in Figure A1. Here, we see that an exceedance probability of 0.001 corresponds to an EIDS of 0.20 inch (0.508 mm). This value is similar to that suggested in [8] for AM parts with as-built surfaces. A discussion on the introduction of the concept of an exceedance probability curve is given in [40].

## Appendix B. The Stress Intensity Factor Correction for the Tu-Delft Crack Growth Data

This paper has made use of a dataset of the crack growth data published in [30]. The raw data from that publication are also available online at [41]. This dataset originally made use of the following stress intensity factor solution for a single-edge notch tension specimen of width W and crack length a:

(A1) $$K=\left(\beta_{Tada}\right)\left(\beta_{FEM}\right)\sigma \sqrt{\pi a}$$

where

(A2) $$\beta_{Tada}=1.12-0.231\left(\frac{a}{W}\right)+10.55{\left(\frac{a}{W}\right)}^{2}-21.72{\left(\frac{a}{W}\right)}^{3}+30.39{\left(\frac{a}{W}\right)}^{4}$$

is the *β* correction factor solution for a single edge notch tension specimen obtained from [42], and

(A3) $$\beta_{FEM}=1.0056-0.5729\left(\frac{a}{W}\right)-0.8072{\left(\frac{a}{W}\right)}^{2}+1.5448{\left(\frac{a}{W}\right)}^{3}-0.2173{\left(\frac{a}{W}\right)}^{4}$$

is a correction factor obtained using the finite element method to account for the aspect ratio of the specimens used in the TUDelft study falling just outside the range covered by the solution for *β_Tad_*_a_.

Unfortunately, the solution for *β_Tada_* obtained from [42], although not explicitly stated in the reference, is for a pinned boundary condition. The authors of [30] misinterpreted it as a solution for the clamped boundary condition matching the condition of their experiments. In the present paper, the authors have accessed the raw data from [41] and reprocessed it using the *β* factor for a clamped single-edge notch tension specimen published in [43] for H/W = 1.5. This *β* factor solution can be expressed as:

*β*(H/W = 1.5) = 2.6745 (a/W)^3^ − 1.3093 (a/W)^2^ + 0.1453 (a/W) + 1.0736. (A4)
